# Cd81 Interacts with the T Cell Receptor to Suppress Signaling

**DOI:** 10.1371/journal.pone.0050396

**Published:** 2012-11-30

**Authors:** Safak Isil Cevik, Nazli Keskin, Serkan Belkaya, Meral Ilcim Ozlu, Emre Deniz, Uygar Halis Tazebay, Batu Erman

**Affiliations:** 1 Biological Sciences and Bioengineering Program, Faculty of Engineering and Natural Sciences, Sabanci University, Istanbul, Turkey; 2 Department of Molecular Biology and Genetics, Bilkent University, Ankara, Turkey; 3 Sabanci University Nanotechnology Research and Application Center- SUNUM, Istanbul, Turkey; Imperial College London, United Kingdom

## Abstract

CD81 (TAPA-1) is a ubiquitously expressed tetraspanin protein identified as a component of the B lymphocyte receptor (BCR) and as a receptor for the Hepatitis C Virus. In an effort to identify trans-membrane proteins that interact with the T-cell antigen receptor (TCR), we performed a membrane yeast two hybrid screen and identified CD81 as an interactor of the CD3delta subunit of the TCR. We found that in the absence of CD81, in thymocytes from knockout mice, TCR engagement resulted in stronger signals. These results were recapitulated in T cell lines that express low levels of CD81 through shRNA mediated silencing. Increased signaling did not result from alterations in the levels of TCR on the surface of T lymphocytes. Although CD81 is not essential for normal T lymphocyte development, it plays an important role in regulating TCR and possibly pre-TCR signal transduction by controlling the strength of signaling. CD81 dependent alterations in thymocyte signaling are evident in increased CD5 expression on CD81 deficient double positive (DP) thymocytes. We conclude that CD81 interacts with the T cell receptor to suppress signaling.

## Introduction

The T cell receptor (TCR) is expressed as a multi-subunit complex on the surface of thymocytes and T lymphocytes, made up of eight polypeptides (TCRαβ, CD3γε, CD3δε, TCRζζ). Immunoreceptor tyrosine based activation motifs (ITAM) in the cytoplasmic tails of these subunits provide a multiplicity of docking sites for recruited signal-transducing proteins. Individual TCR subunits assemble in the endoplasmic reticulum (ER) of T lymphocytes following a defined order, whereby TCRα-CD3δε trimers and TCRβ-CD3γε trimers first assemble into a six chain complex which associates with a dimer of TCRζ subunits, before being exported to the cell surface [1]. Inactivation of the genes encoding individual subunits of the TCR results in an arrest of thymocyte development [2]–[4].

CD3δ deficient thymocytes cannot receive proper TCR signals for positive selection at the CD4+CD8+ double positive (DP) stage; however, pre-TCR signals at the earlier CD4−CD8− double negative (DN) stage are not affected and these early thymocytes can differentiate to the DP stage [5]. Pre-TCR signals do not require the CD4 or CD8 co-receptors for signaling, as none are expressed at the DN stage, while TCR signals at the DP stage are uniquely dependent on co-receptors for positive selection signaling [6]. Thus, CD3δ is uniquely required for αβTCR surface expression and signaling but is dispensable for the function of related multi-subunit receptors (pre-TCR and γδTCR). An evolutionarily conserved alpha-CPM motif in the alpha subunit of the TCR is also necessary for positive selection signaling and linking the TCR to the CD8 co-receptor [7]. This CPM may be important for linking the TCRα “side” of the TCR to CD3δε dimers, while a TCR Cβ FG loop on the TCRβ “side” may be important for communicating with CD3γε dimers [1], [8]. The co-requirement for CD3δ, the TCRα CPM and co-receptors for positive selection signaling indicates that CD3δ may be the link between co-receptors and the TCR [9].

Here we specifically tried to identify membrane proteins that interact with the CD3δ subunit of the TCR. To do so, we used a membrane yeast two hybrid system in which murine CD3δ was expressed as a bait protein in yeast membranes. In T lymphocytes, TCR subunits do not individually get transported to the plasma membrane; rather, individual subunits are retained in the ER and only fully assembled TCR is expressed on the cell surface [1]. Because our screening strategy only involved the expression of the CD3δ subunit, it is possible that the interactions we identified in yeast cells may be occurring in the ER or other sub-cellular membranes.

Using this screening strategy, we identified various membrane proteins that play a role in TCR assembly and signaling. Prime among these molecules was CD81 (TAPA-1), which is a ubiquitously expressed tetraspanin protein [10]. CD81 has been identified as a component of the B lymphocyte receptor and as a receptor for the Hepatitis C Virus [11]–[13]. We chose to explore the role CD81 plays in TCR signaling because of previous reports of its association with the CD4 and CD8 co-receptors [14]. Other reports indicated that upon superantigen exposure, CD81 co-localized with CD3 at the c-SMAC in the immune synapse formed between T and B lymphocytes [15]. Two independent groups generated CD81 deficient mice where redundancy between CD81 and its close homolog CD82 or other tetraspanin proteins, likely resulted in no observable phenotype [16], [17]. While CD81 deficient mice were originally found not to have an *in vivo* T lymphocyte development defect, we find here that developing thymocytes receive stronger signals than WT counterparts, resulting in an *in vivo* upregulation of the CD5 activation marker at the DP thymocyte stage. Indeed, similar to earlier *in vitro* studies which found that CD81 deficiency resulted in enhanced T cell proliferation, in this study we document that CD81 deficient T lymphocytes respond better to antibody mediated signaling.

## Results

### Identification of CD3δ interaction partners by membrane yeast two hybrid screening

In the present study we tried to identify membrane proteins that participate in signal transduction by the T lymphocyte receptor (TCR). To this end, we performed a novel membrane based yeast two hybrid screen by using the CD3δ subunit of the TCR as bait. This screening system allowed us to identify membrane anchored or cytoplasmic proteins that interact with this TCR subunit by reconstituting a functional ubiquitin protein. Formation of functional ubiquitin in turn released a membrane anchored LexA-VP16 transcription factor into the yeast nucleus, that enabled us to select *Saccharomyces cerevisiae* cells which contained interacting bait and prey proteins (**Figure S1**). For screening, we expressed C-terminal ubiquitin fused CD3δ proteins, N-terminally fused to a yeast leader peptide (for membrane insertion) from a bait plasmid, pBT3SUC-CD3δ and an N-terminal ubiquitin fused prey cDNA library derived from human Jurkat T cell lines.

The yeast two hybrid screen was designed with 3 readouts: growth of auxotrophic yeast cells on medium lacking histidine and adenine and the expression of the LacZ gene. We identified 426 colonies on selective plates which also tested positive in a β-galactosidase filter lift-off assay. We reconfirmed positive interactors by independently transforming bait and prey protein encoding plasmids into yeast cells grown on selective medium. The identities of cDNAs identified from positive interactors in independent testing are shown in **Table 1**.

**Table 1:** The identity of cDNAs identified from positive interactors.

CLONE ID	BLAST RESULT	GENE ID
3A3a	NM_001040200.1	Homo sapiens claudin domain containing 1 (CLDND1), transcript variant 7, mRNA.
1G6/1I8a/3I3	NM_000873.2	Homo sapiens intercellular adhesion molecule 2 (ICAM2), mRNA
1C8	NM_002208.3	Homo sapiens integrin, alpha E (ITGAE), mRNA.
1E2b/1E7	NM_021227	Homo sapiens DC2 protein (DC2), mRNA
1G4	NM_018845.1	Homo sapiens recombination activating gene 1 activating protein 1 (RAG1AP1), mRNA
3A2b	NM_004221.4	Homo sapiens interleukin 32 (IL32), transcript variant 2, mRNA.
3A5b	NM_025124.1	Homo sapiens transmembrane protein 134 (TMEM134), mRNA.
3A7b	NM_003329.2	Homo sapiens thioredoxin (TXN), mRNA
3B3b/3K8	NM_032927.2	Homo sapiens transmembrane protein 128 (TMEM128), mRNA
3C1	NM_005745.6	Homo sapiens B-cell receptor-associated protein 31 (BCAP31), mRNA
3I1	NM_004356.3	Homo sapiens CD81 molecule (CD81), mRNA
1G1	NM_198793.2	Homo sapiens CD47 molecule (CD47), transcript variant 2, mRNA
1H4/3E1a	NM_144638.1	Homo sapiens transmembrane protein 42 (TMEM42), mRNA
1J8A/1K1a/1K1b	NM_014051.2	Homo sapiens transmembrane protein 14A (TMEM14A), mRNA

### Specificity of CD3δ interactions in yeast cells expressing TCR subunits

Because the CD3δ subunit of the TCR is known to hetero-dimerize with the CD3ε subunit in T lymphocytes, we tested the specificity of interaction in yeast cell membranes by confirming that yeast cells expressing CD3δ fused to the C-terminal domain of ubiquitin (CD3δ-Cub) and CD3ε fused to the N-terminal domain of ubiquitin (CD3ε-Nub) could indeed grow on selective media. While yeast cells expressing CD3δ-Cub only, or CD3δ-Cub together with an unrelated prey protein did not grow, yeast cells expressing CD3δ-Cub and CD3ε-NubG grew and generated many colonies on selective plates. Next, we tested whether the interactions of TCR subunits identified in yeast membranes are similar to those in mouse T lymphocytes, by assessing the interaction of CD3δ-Cub with TCRα-NubG or TCRβ-NubG. In mouse DP thymocytes, CD3δ forms a heterodimer with CD3ε, and then associates with TCRα in a TCRα-CD3δ-CD3γ trimer and CD3δ does not directly homodimerize with TCRα or TCRβ [1]. In CD3δ-Cub + TCRα-NubG, or CD3δ-Cub + TCRβ-NubG expressing yeast cells grown on Leu-,His-,Trp-,Ade- selective plates, the number of colonies obtained was either zero or very few, compared to yeast cells expressing interacting CD3δ-Cub and CD3ε-NubG proteins (**Table 2**). We conclude that the split ubiquitin yeast expression system can recapitulate the membrane environment that allows TCR subunits to interact with each other, and continued to analyze the proteins encoded by the Jurkat T lymphocyte library that interact with CD3δ-Cub. We decided to further investigate the functional significance of the interaction between CD3δ and a clone expressing a CD81 cDNA, which was isolated from the Jurkat T lymphocyte library and scored as a positive interactor in our secondary screen (**Table 1**).

**Table 2:** CD3δ interacts with TCR subunits in *S. cerevisiae*.

Prey	−L	−T	−LT	−LTH	−LTHA
Positive Control	+	+	+	+	+
Negative Control	+	+	+	−	−
Empty Prey	+	+	+	−	−
TCRα	+	+	+	−	−
TCRβ	+	+	+	+/−	+/−
CD3δ	+	+	+	+/−	+/−
CD3ε	+	+	+	+	+

Auxotrophic yeast strains transformed with a bait plasmid encoding CD3δ carrying a Leu2 gene and a prey plasmid encoding a Trp2 gene. Yeast cells transformed only with a bait plasmid or only with a prey plasmid or with bait and prey plasmids together could grow on Leu- or Trp- or Leu-,Trp- plates respectively. Growth on Leu-,His-,Trp-, or Leu-,His-,Trp-,Ade- selective plates required the interaction of the CD3δ bait with the indicated prey cDNA's. + denotes growth of >100 colonies and +/− denotes growth of <10 colonies on the indicated plates.

### Confirmation of the interaction between CD3δ and CD81 in human cell lines

**Figure 1:**
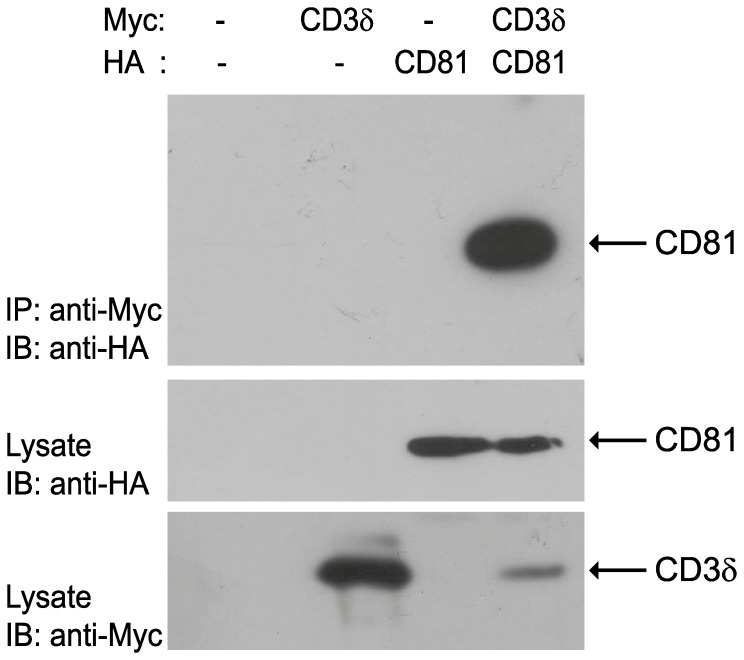
CD81 interacts with CD3δ. HEK293T cells were transfected with plasmids encoding CD81 protein in the presence (lane 4) or absence of CD3δ (lanes 3). Top row: Lysates from untransfected HEK293T cells (lane 1) or transfected cells (lanes 2–4) were immunoprecipitated with anti-Myc antibodies and immunoblotted with anti-HA antibodies. Anti-HA immunoblot shows the presence of a 26 kD band corresponding to the CD81 protein co-immunoprecipitated with Myc-CD3δ protein. Middle row: anti-HA immunoblot of lysates demonstrates the expression of CD81 specifically in cells transfected with this construct (lane 3–4). Bottom row: anti-Myc immunoblot of lysates demonstrates the expression of CD3δ in lanes 2 and 4.

We assessed whether we could recapitulate the CD3δ and CD81 interaction in mammalian cells by co-transfecting human HEK293T cells with expression plasmids encoding C-terminal Myc-epitope tagged CD3δ proteins and N-terminal HA-epitope tagged CD81 proteins. Transfected cell lysates containing CD3δ only, CD81 only, or both proteins together were immunoprecipitated with anti-Myc epitope specific antibodies and blotted with anti-HA specific antibodies, revealing a specific interaction between these proteins in human cell lines (**Figure 1**). Notably, as HEK293T cells do not express the rest of the TCR subunits, this interaction between CD3δ and CD81 likely occurs in sub-cellular membranes rather than the plasma membrane, similar to the interactions we observed in yeast cells.

We further wanted to test the association between CD3δ and CD81 in a setting where CD3δ could assemble into a TCR and be expressed on the cell surface. To this end, we expressed all subunits of the TCR in multi-cistronic expression plasmids in the presence or absence of epitope tagged CD3δ and CD81 in HEK293T cells. We first assessed the cell surface expression of the assembled TCR by flow cytometry and found that up to 40% of transfected cells could indeed express surface TCR complexes containing TCRβ (**Figure S2A**). The assembly and surface expression of TCR in HEK293T cells was specific, as cells expressing only TCRα and TCRβ or only the CD3 subunits CD3δ, CD3ε, CD3γ, TCRζ could not express detectible levels of TCRβ on the cell surface. We next tested whether epitope tagged CD3δ subunits could assemble into multi-subunit TCR complexes. In fact, immunoprecipitation with anti-Myc epitope specific antibodies followed by anti-TCRα blotting revealed that Myc-CD3δ could be found in complexes which contained TCRα (data not shown). Finally we assembled TCR in HEK293T cells and assessed the binding of HA-epitope tagged CD81 with Myc-epitope tagged CD3δ in these cells. Similar to the CD81-CD3δ interaction we previously observed in the absence of full TCR, CD3δ could interact with CD81 in the presence of TCR assembled in HEK293T cells (**Figure S2B**). Thus, CD3δ can interact with CD81 either in the presence or absence of fully assembled TCR.

### Functional significance of the CD3δ-CD81 interaction

**Figure 2:**
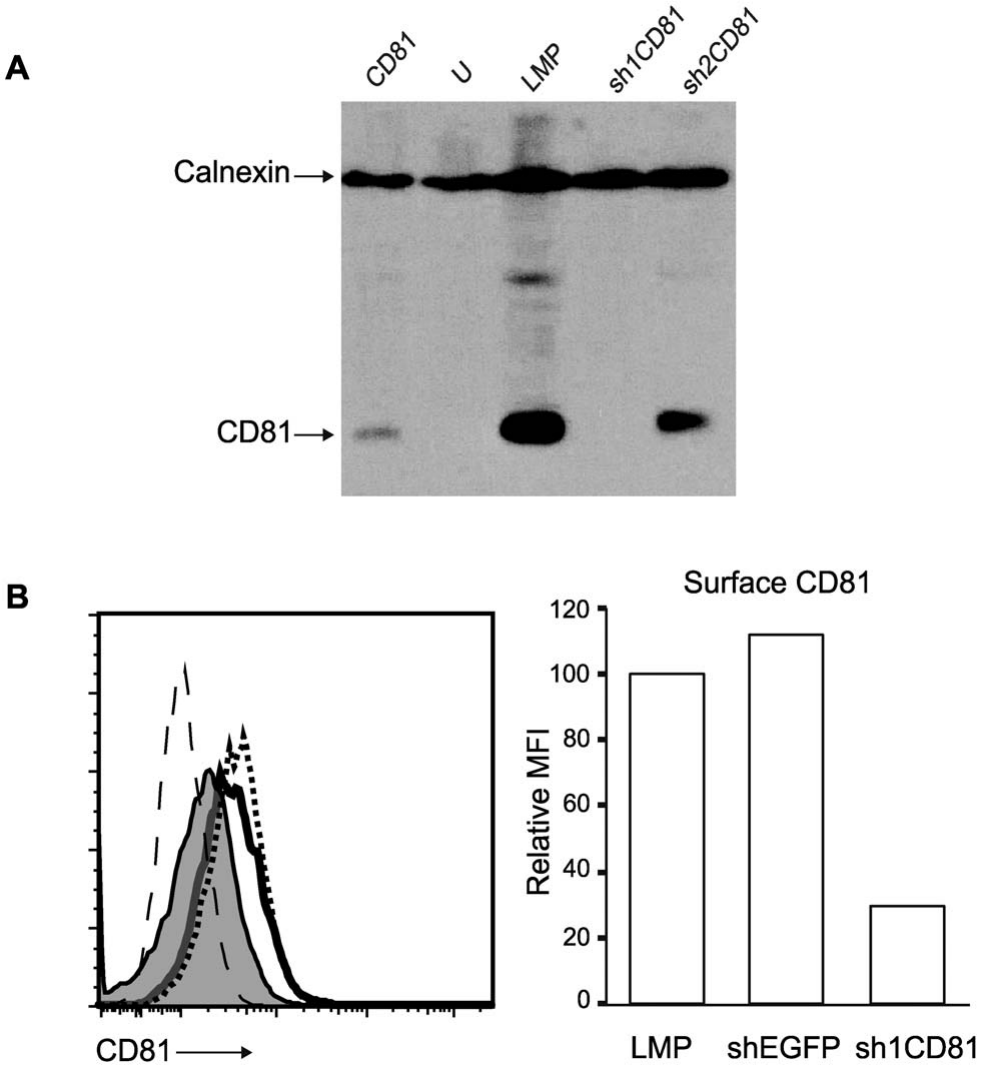
CD81shRNAs knock down CD81 protein expression and surface CD81 expression. (**a**) HA-CD81 transfected HEK293T cells with or without shRNA expression were solubilized in Triton X-100 detergent and resolved by reducing SDS-PAGE and blotted with anti-HA Ab. Lysates from HEK293T cells expressing HA-CD81 alone (lane1), untransfected cells (lane 2), cells expressing HA-CD81 plus empty pLMP shRNA expression construct (lane 3) and HA-CD81 plus sh1CD81 or sh2CD81 expressing pLMP constructs (lanes 4 and 5 respectively) were blotted with anti-HA Ab revealing a 26 KDa HA tagged CD81 band. Equal loading was confirmed by blotting the same membrane with anti-calnexin Ab revealing a 90 KDa calnexin band. (**b**) NIH3T3 cells infected with retrovirus coding shRNA against EGFP (shEGFP) (heavy dotted histogram), empty pLMP shRNA expression construct (solid histogram) or sh1CD81 expressing pLMP construct (tinted histogram) were stained with anti-CD81 biotin followed by streptavidin-PE. Histograms show surface CD81 levels of the cells. The relative mean fluorescence intensity (MFI) normalized to CD81 expression on empty LMP transfected NIH3T3 cells (set to 100) is plotted as a bar graph.

Because the CD3δ subunit of the TCR and the tetraspanin protein CD81 could interact in membrane environments which mimicked the T lymphocyte membrane, we wanted to determine whether the interaction between the CD3δ and CD81 has a functional significance for TCR assembly and signaling. To this end, we decided to downregulate CD81 expression in cell lines by shRNA mediated expression arrest. To screen for functional shRNAs, we overexpressed HA-epitope tagged CD81 cDNAs along with two different shRNAs against different regions of the CD81 cDNA in HEK293T cells and found that CD81 protein levels were dramatically downregulated by one of these shRNAs (sh1CD81), while CD81 levels did not decrease in cells co-expressing an empty shRNA expression plasmid (**Figure 2A**).

To further assess whether the shRNA specific against the CD81 cDNA could downregulate cell surface CD81 levels expressed from endogenous genes, we generated retroviruses expressing shRNA either against a non-specific EGFP protein or against CD81 (sh1CD81), and infected the mouse NIH 3T3 cell line with these retroviruses. Analysis by flow cytometry revealed that the surface expression of endogenous CD81 proteins were dramatically reduced in cells infected with retroviruses expressing sh1CD81 but not with shRNA against EGFP (**Figure 2B**).

**Figure 3:**
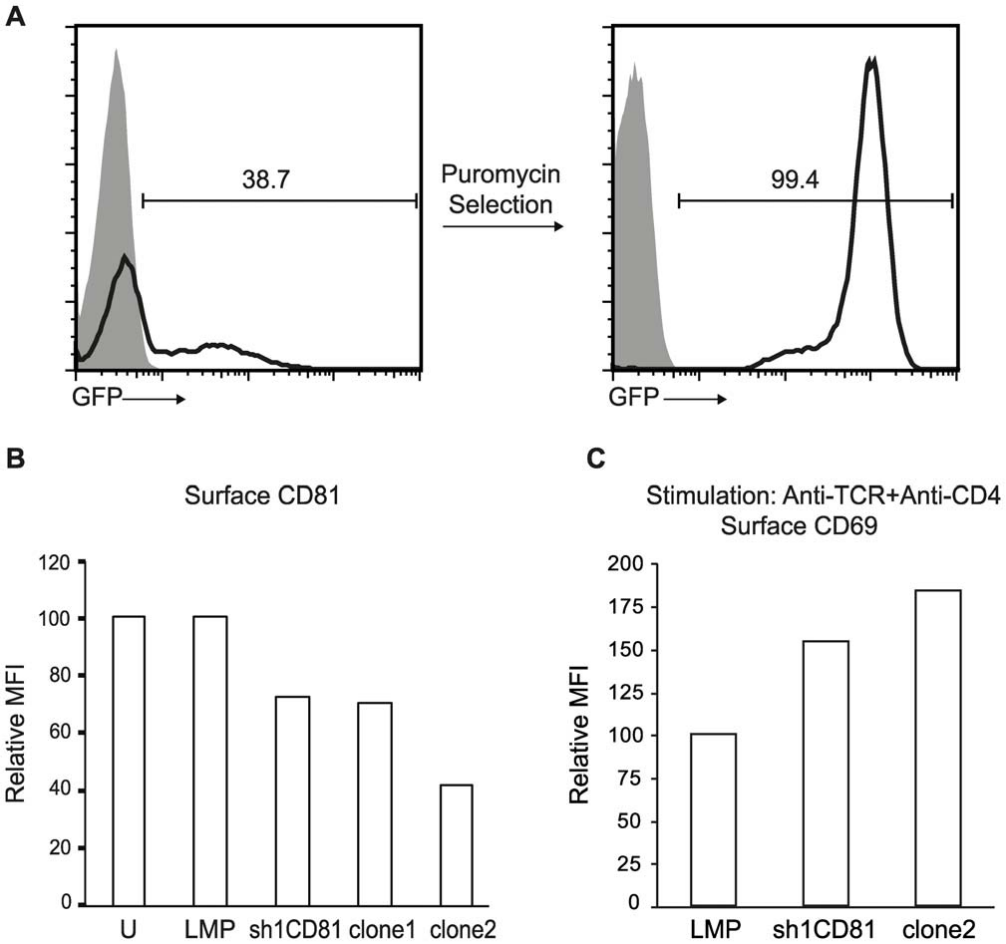
Stable expression of sh1CD81 downregulates surface CD81 expression and increases TCR mediated activation. (**a**) GFP expression before and after puromycin selection in VL3.3M2 cell lines transfected with pLMP encoding CD81 shRNA (sh1CD81) shows the percentage of cells expressing CD81 shRNA. Shaded histograms show fluorescensce of untransfected VL3.3M2 cells and black lined histograms show GFP expression in transfected and antibiotic selected cells. (**b**) CD81 shRNAs decrease surface CD81 expression in stably transfected VL3.3M2 cells. Relative MFI of CD81 expression on the surface of VL3.3M2 cells that are untransfected (U), or stably transfected with empty pLMP constructs (LMP) or with pLMP-sh1CD81 constructs (sh1CD81) or single cell cloned stable sh1CD81 expressing clones (clone1 and clone2) was determined by flow cytometry and plotted as bar graphs. Surface CD81 expression of untransfected VL3.3M2 cells was set to 100. (**c**) Relative MFI of surface CD69 expression on VL3.3M2 cells stimulated with plate bound anti-TCRß (1 µg) and anti-CD4 (1 µg). Surface CD69 expression of empty pLMP transfected VL3.3M2 cells was set to 100.

To test the function of CD81 in T lymphocytes, we transfected the murine CD4+CD8+ double positive VL3.3M2 cell line with plasmids expressing sh1CD81, the shRNA previously determined to be capable of silencing CD81 expression. The expression construct for sh1CD81 also expressed a puromycin resistance gene and an EGFP marker, so we identified stably transfected cells in the presence of the antibiotic puromycin and followed CD81 expression in these cells by EGFP expression. After antibiotic selection, VL3.3M2 cells were uniformly EGFP positive (**Figure 3A**). Furthermore, compared to untransfected cells or those stably expressing puromycin and EGFP without sh1CD81 (LMP), sh1CD81 expressing VL3.3M2 cells downregulated surface CD81 approximately 30% as measured by the mean fluorescence intensity of surface bound anti-CD81 antibodies in a flow cytometer. In order to generate T cell lines that further downregulated surface CD81 expression, we generated single cell clones from this pool of sh1CD81 expressing VL3.3M2 cells, by limiting dilution. We identified two clones which further downregulated surface CD81 expression up to 60% (**Figure 3B**).

To ensure that sh1CD81 expression specifically silenced CD81 and not other surface T cell markers involved in TCR signal transduction, we assessed surface TCR levels in either the stably transfected pools or in the individual clones by anti-TCRβ staining (**Figure S3A**). Compared to the TCR levels in either untransfected VL3.3M2 cells, or those transfected by empty shRNA expression plasmids (LMP), stable transfected VL3.3M2 cells which expressed sh1CD81 and silenced CD81 did not downregulate surface TCR expression. Thus, silencing CD81 in VL3.3M2 thymocytes specifically downregulates surface CD81 expression without altering surface TCR levels. On the other hand when we overexpressed a human CD81 cDNA in either HEK293T cells with a reconstituted TCR, or in murine VL3.3M2 cells, we observed a significant reduction in surface TCR expression (data not shown). Thus overexpression of CD81 interferes with TCR assembly while decreasing CD81 expression by shRNA mediated knockdown has no effect on this process.

To identify the role of CD81 in TCR signaling, we stimulated stably transfected sh1CD81 expressing VL3.3M2 cells with different levels of surface CD81, with anti-TCRβ plus anti-CD4 mAb. We assessed the intensity of TCR signaling upon antibody crosslinking by CD69 upregulation. When we stimulated untransfected VL3.3M2 thymocytes, (sh1CD81) stable transfected pools or clone2, expressing high, intermediate or low levels of CD81 respectively, clone 2 responded by upregulating dramatically more CD69, compared to either the pool of sh1CD81 silenced VL3.3M2 cells or untransfected cells (**Figure 3C** **and Figure S3B**). Dramatically, we noticed an inverse correlation between the level of surface CD81 expression and CD69 upregulation upon anti-TCRβ and anti-CD4 mAb crosslinking. Thus, we found that downregulating surface CD81 expression increases the signaling intensity of CD4 co-receptor dependent TCR in DP VL3.3M2 thymocytes.

**Figure 4:**
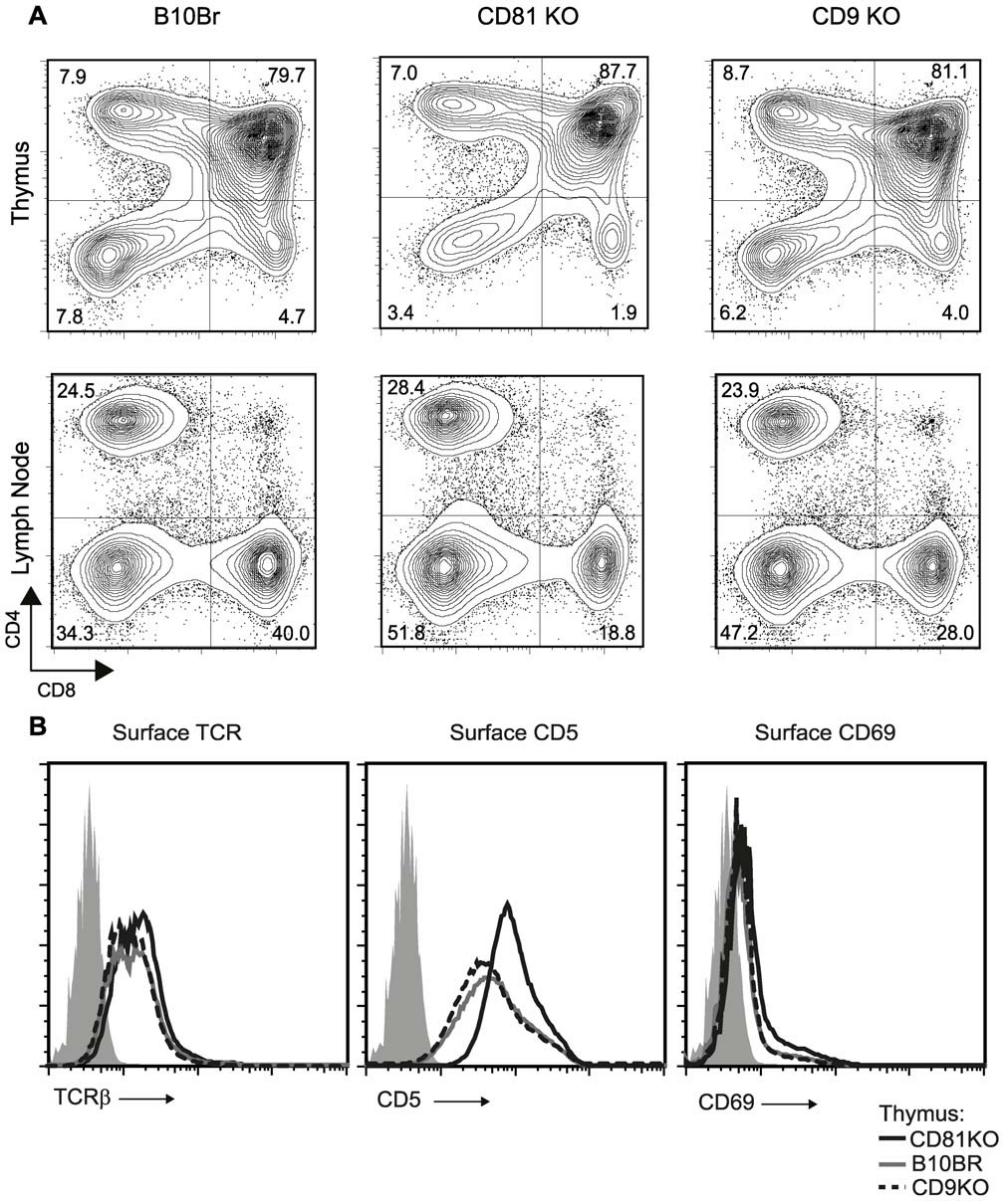
Inreased signaling intensity in CD81−/− thymocytes. (**a**) Deficiency of CD81 or CD9 does not affect T lymphocyte development and thymic cellularity. Thymocytes from B10, CD81−/− and CD9−/− mice were analyzed by multicolor flow cytometry. CD4 vs CD8 contour plots of thymocytes (top row) and lymph node cells (bottom row) are shown. (**b**) CD81 deficiency increases in vivo signaling intensity in DP thymocytes. Single parameter histograms of TCRß, CD5 and CD69 expression on electronically gated CD4+CD8+ DP thymocytes from B10 (dashed line), CD81−/− (black line) and CD9−/− (grey line) are shown. The electronic gate used is depicted as a box in the top panels in (a).

Because CD81 is a tetraspanin protein enriched in the detergent insoluble membrane fraction which was previously shown to interact directly with the CD4 co-receptor in lymphocytes [14] and because we demonstrated in this study that CD81 could in fact interact with CD3δ, a component of the TCR, we hypothesized that CD81 played an important role in TCR signaling, perhaps regulating the migration of the TCR into lipid rafts during signaling. To asses the significance of CD81 in TCR signaling in an *in vivo* setting, we utilized CD81−/− mice and as a control, CD9−/− mice because CD9 is a similar tetraspanin protein. Although the profile of developing thymocytes in these mice were documented in previous studies, the impact of the absence of CD81 on *in vivo* TCR signaling has never been determined [16], [17]. The cellularity and ratios among the four main populations of thymocytes (DN, DP, CD4 and CD8SP) and lymph node cells of either CD81−/− or CD9−/− mice were similar to WT mice (**Figure 4A**). Furthermore, the absence of CD81 or CD9 did not affect the surface expression levels of TCR on developing thymocytes, mimicking our results in VL3.3M2 thymocyte cell lines in which we downregulated CD81 expression by shRNA mediated expression arrest (**Figure 4B**).

Dramatically, the expression of CD5 on *ex vivo* DP thymocytes from CD81−/− mice was significantly increased compared to DP thymocytes from WT and CD9−/− mice, indicating that although CD81 and CD9 can interact with each other only CD81 can inhibit *in vivo* TCR signaling (**Figure 4B**). We also assessed the levels of CD69 on *ex vivo* DP thymocytes from WT, CD81−/− or CD9−/− mice and found that this marker of recent thymocyte activation was indistinguishable between these three populations. Because CD5 expression levels on DP thymocytes directly reflect the intensity of *in vivo* TCR signaling, we conclude that CD81 is a negative regulator of TCR signaling [18]–[21]. We confirmed that the CD81−/− mice indeed lacked surface CD81 expression while retaining surface CD9 expression on thymocytes and lymphocytes (**Figure S4A and S4B**). Furthermore we assessed the expression of TCR, CD5 and CD69 on CD4+ or CD8+ thymocytes (**Figure S4C**). While the expression of these markers between WT and CD81−/− CD4+ thymocytes were indistinguishable, we observed that pre-DP, immature CD8+ single positive (ISP) cells, characterized by low TCR and CD5 expression, were lacking in CD81−/− mice. Thus in the absence of CD81 inhibition, thymocytes progress faster through development.

**Figure 5:**
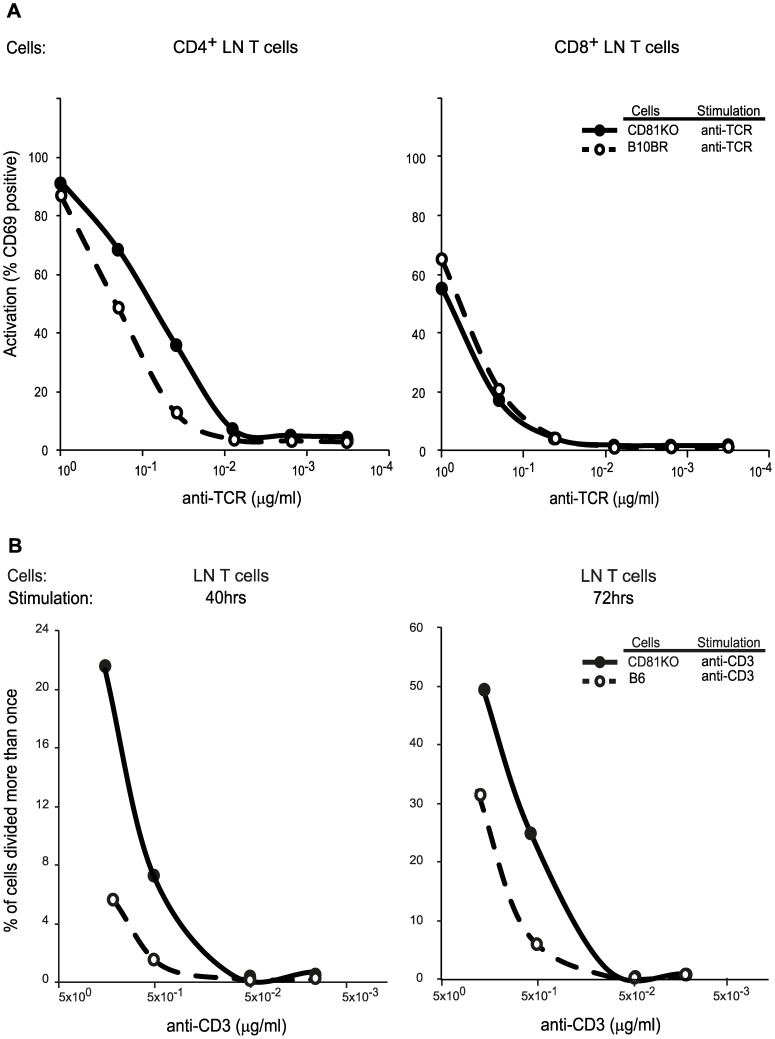
CD81 deficiency increases in vitro TCR signaling intensity in CD4+ lymph node T cells. (**a**) Deficiency of CD81 results in more cells that upregulate CD69 upon TCR signaling. Purified LN T cells from B10 or CD81−/− mice were assessed for anti-TCR+anti-CD4 induced up-regulation of CD69 expression. Left panel: After overnight stimulation with plate bound anti-TCR antibodies, electronically gated CD81−/− CD4 LN T cells (filled circles) upregulated dramatically more CD69 compared to WT B10 CD4 LN T cells (open circles). Right panel: Stimulation with anti-TCR did not result in a difference between CD81−/− and WT B10 CD8 LN T cells. (**b**) Frequency of proliferated (>1 cell division) cells after stimulation of CFSE-labelled purified LN CD4+ cells from B6 and CD81−/− mice. Plots of CFSE dye dilution in CD4 T cells stimulated with plate bound anti-CD3 antibodies indicate the frequency of cells with >1 division. Left plot shows proliferation after 40 hr of stimulation and right plot shows proliferation after 72 hr of stimulation. At least two mice in each group were analyzed.

In order to identify the role of CD81 in TCR signaling on lymph node T lymphocytes, we purified these cells from either WT or CD81−/− mice and assessed the upregulation of CD69 upon anti-TCR crosslinking on electronically gated CD4+ or CD8+ lymphocytes (**Figure 5A**). Compared to WT lymphocytes (B10BR), CD81−/− cells responded more robustly to anti-TCRβ crosslinking by upregulating CD69. Specifically, we found a higher percentage of CD69 positive cells at each concentration of stimulating anti-TCRβ antibody when we compared CD81−/− lymphocytes to WT lymphocytes. This effect was specific to CD4 T lymphocytes, as similar numbers of CD8 T lymphocytes from CD81−/− and WT mice were signaled to become CD69 positive. Thus, stimulation of the TCR in the absence of CD81 can generate stronger signals than when CD81 is present, indicating that CD81 normally inhibits TCR signaling.

We next tested whether the lack of CD81 effected events further downstream of TCR signaling. One of the outcomes of anti-TCR stimulation of LN T lymphocytes is their proliferation. Thus we tested whether CD81 deficient lymphocytes proliferated better as a result of increased TCR signaling, compared to WT lymphocytes from C57Bl/6 mice. We purified CD4+ lymph node T lymphocytes, labeled them with the dye CFSE and measured dye dilution upon proliferation of cells stimulated by plate bound anti-CD3 antibodies (**Figure 5B** **and S5**). As compared to lymphocytes from C57Bl/6 mice, lymphocytes from CD81−/− mice proliferated faster as measured by the percentage of cells that lose CFSE dye after 40 and 72 hours of stimulation. The proliferative advantage of CD81−/− CD4+ lymph node T lymphocytes was more dramatic when stimulated with low doses of cross-linking anti-CD3 antibody. Thus, limiting TCR signals and the signaling events that result in CD4+ lymphocyte proliferation are inhibited by CD81.

## Discussion

T lymphocyte signaling is initiated by the engagement of the T cell receptor complex (TCR) and co-receptors (CD4 or CD8) on the cell surface of T lymphocytes and cognate antigen presented on MHC molecules by antigen presenting cells. This engagement results in the phosphorylation of the TCR signaling subunits and activation of downstream signaling pathways. In the current study we focused on the CD3δ signaling subunit of the TCR and identified the tetraspanin membrane protein CD81 as an interaction partner. CD3δ is required for positive selection signaling at the DP stage of thymocyte development. The lack of mature SP T lymphocytes in CD3δ−/− mice indicates that the CD3δ subunit is required either for TCR signaling, or for the expression of this receptor on the surface. On the other hand, unlike the other CD3 subunits, the CD3δ subunit is unique because pre-TCR signaling controlling the DN to DP transition can happen in its absence.

In order to identify putative interaction partners of the CD3δ subunit of TCR, we performed a novel membrane based yeast two hybrid screen. Classical yeast two hybrid screens are limited to identifying interactions between soluble proteins. However, split ubiquitin technology allows for the identification of interactions between membrane anchored bait and prey proteins. In this system, auxotrophic yeast reporter gene activation was driven by the translocation of a transcription factor from the yeast cell plasma membrane to its nucleus. Our bait, CD3δ was targeted to yeast membranes using a SUC2 yeast signal peptide. Because we conducted our yeast two hybrid screen in yeast cells only expressing the CD3δ subunit and not a full TCR, we predicted that this protein would be retained in the ER in agreement with previous studies showing that individual TCR subunits which assemble into full TCR complexes are retained in the ER [22]. We also demonstrate that CD3δ and CD81 could interact in mammalian cell lines by co-immunoprecipitation. In order to test the association between CD3δ and CD81 in a setting where CD3δ could assemble into a TCR and be expressed on the cell surface, we expressed all subunits of the TCR in HEK293T cells. CD3δ continued to interact with CD81 in this cellular setting.

We observed that over-expression of CD81 in either HEK293T cells expressing TCR or in murine CD4+CD8+ double positive VL3.3M2 cells resulted in the downregulation of surface TCR expression. This indicates that either CD81 can interact with TCR subunits in the ER to regulate assembly and surface expression, or that elevated levels of CD81 results in the internalization of surface TCR. While CD81 is clustered to tetraspanin enriched microdomains in the cell surface, it also plays a role in the ER. In fact, in B lymphocytes, the absence of CD81 prevents the exit of the BCR component CD19 to the B lymphocyte cell surface [16], [17], [23]. Unlike the role of CD81 in B lymphocytes, in the current study we find that CD81 overexpression in T cells reduces surface TCR expression, while both shRNA mediated silencing or genetic knockout of CD81 does not affect surface TCR. These findings indicate that CD81 and possibly other tetraspanin proteins function in the ER by regulating the assembly and surface export of multisubunit receptors in lymphocytes.

CD81 is a tetraspanin molecule known to function as a specific membrane dock for signaling proteins in lipid rafts [10]. Antibody mediated stimulation of T lymphocytes indicates that CD81 is an *in vitro* co-stimulator of the TCR [24], [25], suggesting that co-engagement of CD81 with antigen receptors facilitates the reorganization of the membrane, thereby reducing the threshold of cell activation. In the current study, we explored whether CD81 cell surface expression levels affect TCR mediated stimulation. Both in T cell lines and in primary murine T lymphocytes, lower or absent surface CD81 expression resulted in increased TCR stimulation as measured by CD69 upregulation. The increase in TCR signaling in the absence of CD81 in primary lymphocytes was specific to CD4 SP cells, indicating that the influence of tetraspanin enriched domains on TCR signaling may differ in different lymphocyte populations. We infer from this finding that CD81 proteins in tetraspanin enriched microdomains may be limiting the access of the TCR to CD4 coreceptors and associated signaling kinases. CD81 shRNA expressing VL3.3M2 cells continued to express surface CD81, albeit at lower levels compared to WT cells. In these cells anti-CD4 and anti-TCR antibody crosslinking could indeed stimulate CD81^low^ shRNA^+^ cells better than WT VL3.3M2 cells, a finding consistent with the faster migration of the TCR into the detergent insoluble membrane fraction under reduced CD81 expression.

To assess the significance of CD81 in TCR signaling in an *in vivo* setting, we utilized CD81−/− mice and as a control, CD9−/− mice because CD9 is also a tetraspanin with a high similarity to CD81. The absence of CD81 had no effect on the cellularity of the lymphoid organs and the ratios between CD4 and CD8 thymocyte populations and lymph node T cells. The most striking effect of the absence of CD81 was on the levels of CD5 on *ex vivo* DP thymocytes. As CD5 levels on DP thymocytes directly reflect the intensity of *in vivo* TCR signaling, we conclude that CD81 is a negative regulator of TCR signaling. This finding is consistent with increased numbers of CD69 positive CD81−/− lymphocytes compared to WT cells in *in vitro* antibody crosslinking experiments. A role for CD81 in early development of lymphcytes in the thymus has been proposed [26].

We also tested the effects of the absence of CD81 on the proliferation properties of signaled lymphocytes. We found that CD81−/− CD4+ lymph node T cells initiated their proliferation response to anti-CD3 crosslinking faster when compared to WT lymphocytes. A previous study found that after 7 days of anti-CD3 stimulation with anti-CD28 co-stimulation, CD81−/− lymphocytes proliferated as efficiently as WT lymphocytes [27]. In contrast to those studies, our stimulation experiments were conducted for 40 and 72 hours without co-stiumulation, where we observed a dramatic proliferative advantage of CD81 deficient lymphocytes, when TCR signaling initiated. Thus, in the absence of CD81, lymphocytes can proliferate better if TCR signals are limiting. In the current study we identify CD81 as an interactor of TCR subunits and find that TCR signaling on DP thymocytes and SP lymphocytes is inhibited by CD81 molecules.

## Materials and Methods

### Split-ubiquitin Yeast Two-hybrid Screening

The split-ubiquitin system and human Jurkat thymus cDNA libraries were purchased from Dualsystems Biotech (Zurich, Switzerland). Screening was performed according to manufacturer's protocols (DUALmembrane Kit 3). Briefly, to create the CD3δ bait construct, the sequence encoding mouse CD3δ protein after the signal peptide was amplified using *Pfu* DNA polymerase (Fermentas) with overhanging restriction sites. The PCR product was cloned in frame and downstream of the SUC2 signal peptide and upstream of the C-terminal half of ubiquitin (Cub) in the vector pBT3-SUC to create pBT3CD3δ, which expresses a SUC2::CD3δ::Cub-LexAVP16 fusion protein and a LEU2 selectable marker. Jurkat thymus cDNA library cloned into pDSLNx plasmid with a TRP1 selectable marker was used as the prey vector. Bait and prey vectors were sequentially transformed into host yeast strain NMY51(*MATa his3_200 trp1–901 leu2–3,112 ade2 LYS2::(lexAop)_4_-HIS3 ura3::(lexAop)_8_-LacZ ade2::(lexAop)_8_-ADE2*) using lithium acetate transformation. Clones encoding proteins that interact with CD3δ were recovered from *TRP1-LEU2-HIS3*-*ADE2-* and *lacZ*- plates. Plasmid DNA was recovered from yeast cells, and retransformed into the NMY51 yeast strain containing the pBT3CD3δ plasmid for a secondary screen. Clone identity was determined by nucleotide sequencing and cDNAs were transferred from the pDSLNx plasmid into the mammalian expression plasmid pHAMex (Dualsytems), in frame with an N-terminal HA epitope tag.

### Plasmids

Full length CD3δ cDNA was amplified with primers containing overhanging restriction sites (Xho I and Hind III) and cloned into the pcDNA3.1MycHIS plasmid (Invitrogen) in frame with an Myc epitope tag. shRNAs against CD81 encoded by an XhoI-EcoRI fragment amplified from a 97 bp long oligonucleotide with two oligonucleotides (mir30for and mir30rev) were cloned into the retroviral plasmid MSCV-LTRmiR30-PIG (LMP) (Openbiosystems). To express TCR in HEK293T cells, we used two plasmids, pMIGII-TCRα (Vα11.1)-P2A-TCRβ (Vβ3)-IRES-GFP and pMIGII-CD3δ-F2A-CD3γ-T2A-CD3ε-P2A-CD3ζ-P2A-IRES-GFP [28] (kindly provided by D. Vignali). The identity of all plasmids were confirmed by DNA sequencing.

### Cell Culture and Transfection

HEK293T cells were cultured in DMEM supplemented with 10% fetal bovine serum (FBS), at 37°C in a humidified, 5% CO2 incubator and transfected using CaPO_4_ precipitation with 10 µg DNA. VL3-3M2 DP thymocyte cell lines (a gift from C. Guidos) [29] were cultured in RPMI supplemented with 10% FBS and transfected by electroporation with 15 µg DNA per 0.5–1×10^7^ cells in 4 mm electroporation cuvettes with a BTX model 600 electroporator (Harvard Apparatus) using the following settings: 0.4 kV, 100Ω, 950 µF. For stable transfection, cells were harvested 48 hrs after the transfection and resuspended in RPMI with 1 µg/ml puromycin and selected in 96 well plates with limiting dilutions.

### Immunoprecipitation and Immunoblotting

10^7^ HEK293T cells transiently transfected with mammalian expression plasmids encoding Myc-CD3δ and HA-CD81 were solubilized in 1% TritonX-100 lysis buffer and immunoprecipitated with 5 µg anti-cMyc (Roche, 11667149001) and 50 µl 1∶1 slurry of protein G-sepharose (GE healthcare) or using anti-c-Myc-agarose conjugates (Sigma IP0020 Kit). Immunoprecipitates were rocked overnight at 4°C, washed three times with 1× PBS, resuspended in SDS sample buffer, boiled, resolved by 13% SDS-PAGE under reducing conditions and transferred to polyvinylidine difluoride (PVDF) membranes. For immunoblotting, membranes were blocked in 1× PBS containing 0.2% Tween-20 and 5% low-fat milk powder (Fluka FL70166) and incubated with anti-HA peroxidase (Roche, 12013819001), washed three times with PBST visualized with a Super Signal West Dura Extended substrate (Pierce).

### Stimulations

CD4+ or CD8+ LN T lymphocytes were obtained by depletion of Ig+ cells using Biomag beads (Qiagen) resulting in cell purities >95%. For anti-TCR and anti-CD4 stimulation, purified lymph node T cells (2×10^6^ cells/ml) or Vl3-3M2 cells (1×10^6^ cells/ml) were plated in 24-well tissue culture plates previously coated (4°C, overnight) with 0–1000 ng/ml anti-CD4 (GK1.5, BD Biosciences) and/or anti-TCRβ (H57-597, BD Biosciences). Stimulation was performed 16–24 hr at 37°C and analyzed by CD69 staining. For CFSE dye dilution assays, CD4 T lymphocytes were purified by negative selection columns (Miltenyi Biotech mouse CD4+ T cell Isolation Kit, 130-095-248), 0.2 million cells were loaded with 0.5 µM CFSE for 8 min at room temperature (Invitrogen V12883) and incubated in 96well plates previously coated (4°C, overnight) with 0–2000 ng/ml anti-CD3 antibody (clone 145-2C11 (BD 553057) for 40–72 hr at 37°C and analyzed by flow cytometry.

### Antibodies and Flow Cytometry

MAbs with the following specificities were used in the present study: For immunofluorescence to assess the cell surface levels: CD5 (53-7.3), FITC or PE-TCRβ (H57-597), PE-CD4 (GK1.5), Cy5-CD8a (53.6-7) biotin-CD81 (Eat2, 559518), PE- CD69 (H1.2F3) and PE-conjugated streptavidin (all from BD Biosciences) and CD8a (CT-CD8a, Caltag). For stimulations: unconjugated TCRβ (H57-597), CD4 (GK1.5). Cells were harvested, stained with fluorochrome-conjugated antibodies by incubating on ice for 30 min and analyzed by flow cytometry on a FACSCanto or FACSCalibur system (Becton Dickinson). Dead cells were excluded by forward light scatter and propidium iodide (PI) or 7-aminoactinomycin D (7-AAD) staining. Analysis was performed using FlowJo 9.5.2 software.

### Mice

B10.Br, CD81−/− [16] and CD9−/− [30] mice in the B10.Br background were bred in the colony of the Experimental Immunology Branch. CD81−/− animals used for proliferation experiments were backcrossed in this colony to the C57Bl/6 background and were compared to C57Bl/6 mice. Animal experiments were approved by the National Cancer Institute Animal Care and Use Committee and were performed in the Experimental Immunology Branch, National Cancer Institute, Bethesda, MD. All mice used in this study were cared for in accordance with National Institutes of Health guidelines.

### Oligonucleotides used in this study

The following oligonucleotides were used for amplification. Restriction enzyme sites used for cloning are shown in bold lettering.

**CD81-3** 5′-TGC TGT TGA CAG TGA GCG CGC AGC CAT TGT GGT AGC TGT CTA GTG AAG CCA CAG ATG TAG ACA GCT ACC ACA ATG GCT GCA TGC CTA CTG CCT CGG A-3′

**CD81-2** 5′-TGC TGT TGA CAG TGA GCG ATG TCA TTA TGA TCT TTG AGA TTA GTG AAG CCA CAG ATG TAA TCT CAA AGA TCA TAA TGA CAG TGC CTA CTG CCT CGG A-3′

**CD81-1** 5′-TGC TGT TGA CAG TGA GCG CGC TGT CAT TAT GAT CTT TGA GTA GTG AAG CCA CAG ATG TAC TCA AAG ATC ATA ATG ACA GCT TGC CTA CTG CCT CGG A-3′

**mir30for** 5′-CAG AAG G**CT CGA G**AA GGT ATA TTG CTG TTG ACA GTG AGC G-3′

**mir30rev** 5′-CTA AAG TAG CCC CTT **GAA TTC** CGA GGC AGT AGG CA-3′

**Sfi-d-F1** 5′-CCG **GGC CAT TAC GGC C**TT CAA GGT ACA AGT GAC CG-3′

**Sfi-d-R** 5′-GAC **GGC CGA GCC GGC C**TT AGA TTT CTT GTT CCG GGG-3′

**FRET-d-F** 5′-CCG **CTC GAG** GCC ACC ATG GAA CAC AGC GGG ATT CTG-3′

**FRET-d-R** 5′-CCC **AAG CTT** AGA TTT CTT GTT CCG GGG-3′

## Supporting Information

Figure S1 **The split ubiquitin membrane yeast two hybrid system.** A bait protein (X) is fused to the C-terminal domain of human ubiquitin protein (Cub) and a LexA/VP16 transcription factor. Interaction of this bait with a prey (Y) encoded by a human Jurkat T cell cDNA library fused to the N-terminal domain of ubiquitin (Nub) results in the reconstitution of ubiquitin activity. The interaction dependent close proximity of Cub and Nub is recognized by yeast ubiquitin specific proteases (UBPs) which cleave the LexA/VP16 domain, setting it free to translocate to the yeast nucleus which results in the expression of the HIS3 and ADE2 auxotrophic markers and LacZ reporter genes controlled by LexA binding sites.
(TIF)

Figure S2 **CD81 interacts with CD3δ in the presence and absence of surface TCR expression.** (**a**) De novo TCR expression in HEK293 cells. HEK293 cells were transfected with two plasmids encoding TCRα+TCRβ and CD3δ+CD3γ+CD3ε+TCRζ and analyzed by flow cytometry at the indicated times after transfection for surface TCR expression by PE conjugated anti-TCRβ staining. Polycistronic expression plasmids contained and IRES-EGFP reporter, and the TCR expression on GFP+ cells is shown. (**b**) CD81 interacts with CD3δ. Combinations of plasmids used for transfection are indicated by (+). NP-40 lysates of transfected cells were prepared 40 hours after transfection (corresponding to peak surface TCR expression) and immunoprecipitated with anti-Myc epitope Ab and blotted for anti-HA epitope Ab. Lysates were also blotted directly with anti-HA epitope Ab to show the expression of HA-CD81 in transfected cells.
(TIF)

Figure S3 **Stable expression of sh1CD81 increases TCR mediated activation without affecting surface TCR expression.** (a) CD81 shRNAs does not affect surface TCR expression in stably transfected VL3.3M2 cells. Relative MFI of TCRβ expression on the surface of VL3.3M2 cells that are untransfected (U), or stably transfected with empty pLMP constructs (LMP) or with pLMP-sh1CD81 constructs (sh1CD81) or single cell cloned stable sh1CD81 expressing clones (clone1 and clone2) was determined by flow cytometry and plotted as bar graphs. Surface TCRβ expression of untransfected VL3.3M2 cells was set to 100. (**b**) Surface CD69 expression activated by anti-TCR+anti-CD4 co-crosslinking is inversely proportional to the level of surface CD81 expression. VL3.3M2 cells were crosslinked with plate bound anti-TCR+anti-CD4 antibodies and MFI of surface CD69 expression on empty LMP transfected (squares), sh1CD81 expressing (circles) and single cell cloned high sh1CD81 expressing clone 2 cells (triangles) were plotted for increasing antibody concentrations.
(TIF)

Figure S4 **Surface expression of CD81, CD9, TCR, CD5 and CD69 on the surface of CD81−/− and CD9−/− thymocytes and lymph node cells.** (a) Surface CD81 and CD9 expression on CD81−/− (black histograms) and CD9−/− (grey histograms) on DP thymocytes shown in the gate defined in Figure 4. Isotype control staining is shown as a shaded histogram. (b) Surface CD81 and CD9 expression on CD81−/− (black histograms) and CD9−/− (grey histograms) on LN cells. (c) Surface TCRβ, CD5 and CD69 expression on CD81−/− (black histograms) and CD9−/− (grey histograms) on CD4 (top row) and CD8 (bottom row) SP thymocytes.
(TIF)

Figure S5 **CD81−/− LN CD4+ lymphocytes proliferate faster than WT cells.** Frequency of proliferated (>1 cell division) cells after stimulation of CFSE-labeled purified LN CD4+ cells from B6 and CD81−/− mice. Histograms show CFSE expression in stimulated CD4 T cells and numbers in the left gate indicate the frequency of cells with >1 division and the numbers in the right gate indicate un-proliferated cells.
(TIF)
