# Supplementary figures and images for: Cd81 Interacts with the T Cell Receptor to Suppress Signaling
Source: PLoS One. 2012 Nov 30;7(11):e50396. doi: 10.1371/journal.pone.0050396 (PMC3511562; doi:10.1371/journal.pone.0050396)

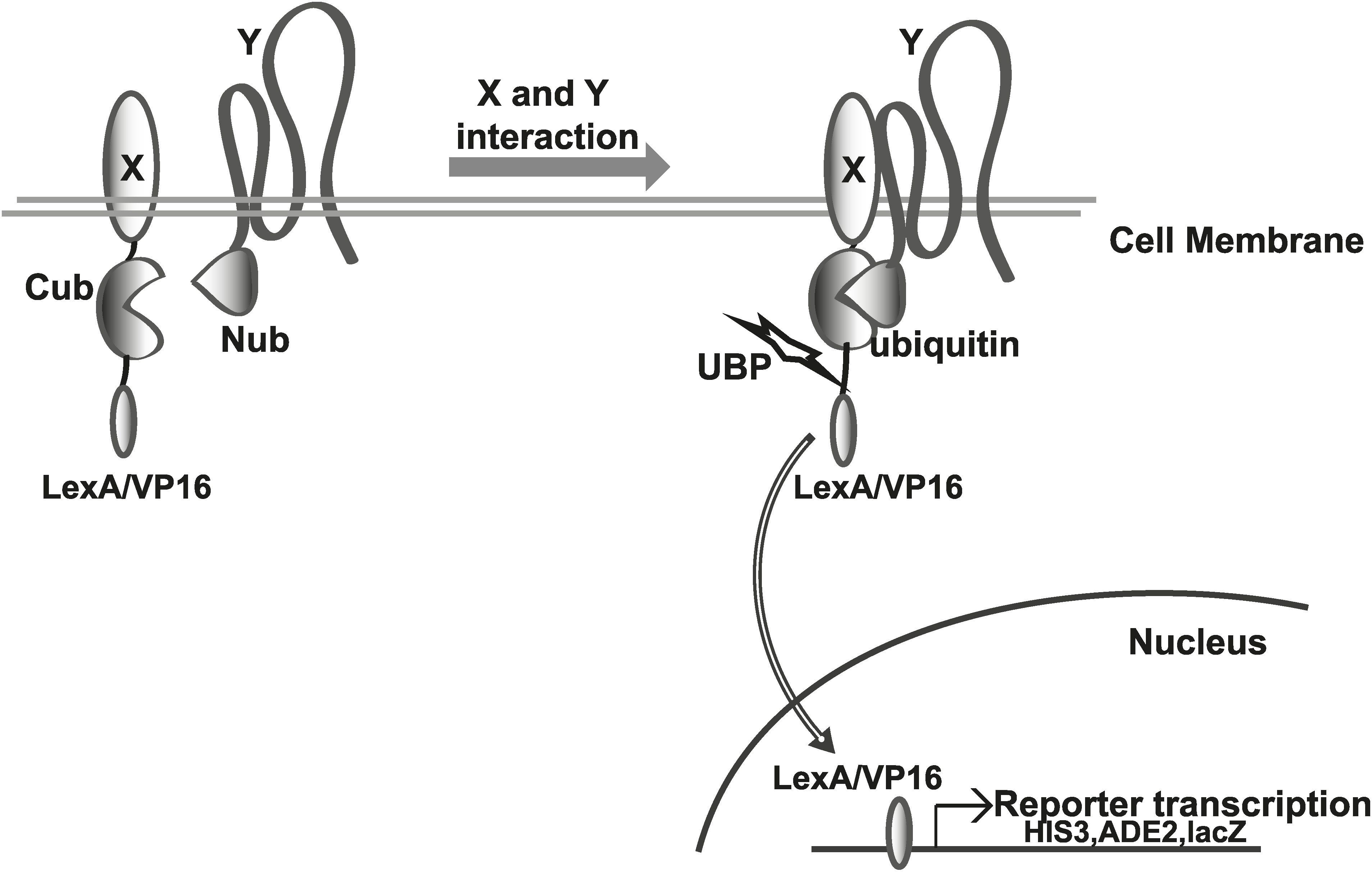

Supplement: Figure S1 — The split ubiquitin membrane yeast two hybrid system. A bait protein (X) is fused to the C-terminal domain of human ubiquitin protein (Cub) and a LexA/VP16 transcription factor. Interaction of this bait with a prey (Y) encoded by a human Jurkat T cell cDNA library fused to the N-terminal domain of ubiquitin (Nub) results in the reconstitution of ubiquitin activity. The interaction dependent close proximity of Cub and Nub is recognized by yeast ubiquitin specific proteases (UBPs) which cleave the LexA/VP16 domain, setting it free to translocate to the yeast nucleus which results in the expression of the HIS3 and ADE2 auxotrophic markers and LacZ reporter genes controlled by LexA binding sites. (TIF) [file pone.0050396.s001.tif]

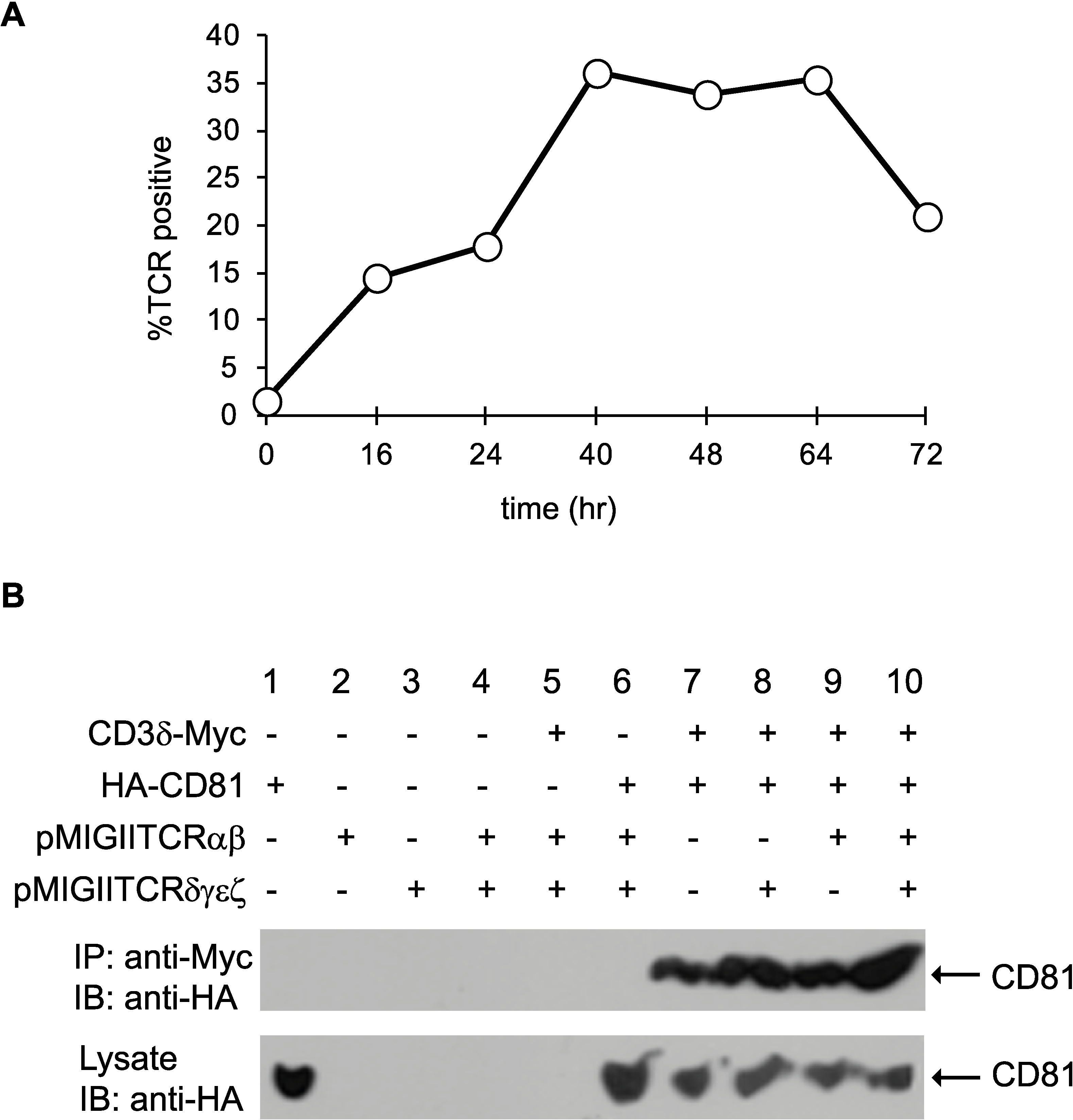

Supplement: Figure S2 — CD81 interacts with CD3δ in the presence and absence of surface TCR expression. (a) De novo TCR expression in HEK293 cells. HEK293 cells were transfected with two plasmids encoding TCRα+TCRβ and CD3δ+CD3γ+CD3ε+TCRζ and analyzed by flow cytometry at the indicated times after transfection for surface TCR expression by PE conjugated anti-TCRβ staining. Polycistronic expression plasmids contained and IRES-EGFP reporter, and the TCR expression on GFP+ cells is shown. (b) CD81 interacts with CD3δ. Combinations of plasmids used for transfection are indicated by (+). NP-40 lysates of transfected cells were prepared 40 hours after transfection (corresponding to peak surface TCR expression) and immunoprecipitated with anti-Myc epitope Ab and blotted for anti-HA epitope Ab. Lysates were also blotted directly with anti-HA epitope Ab to show the expression of HA-CD81 in transfected cells. (TIF) [file pone.0050396.s002.tif]

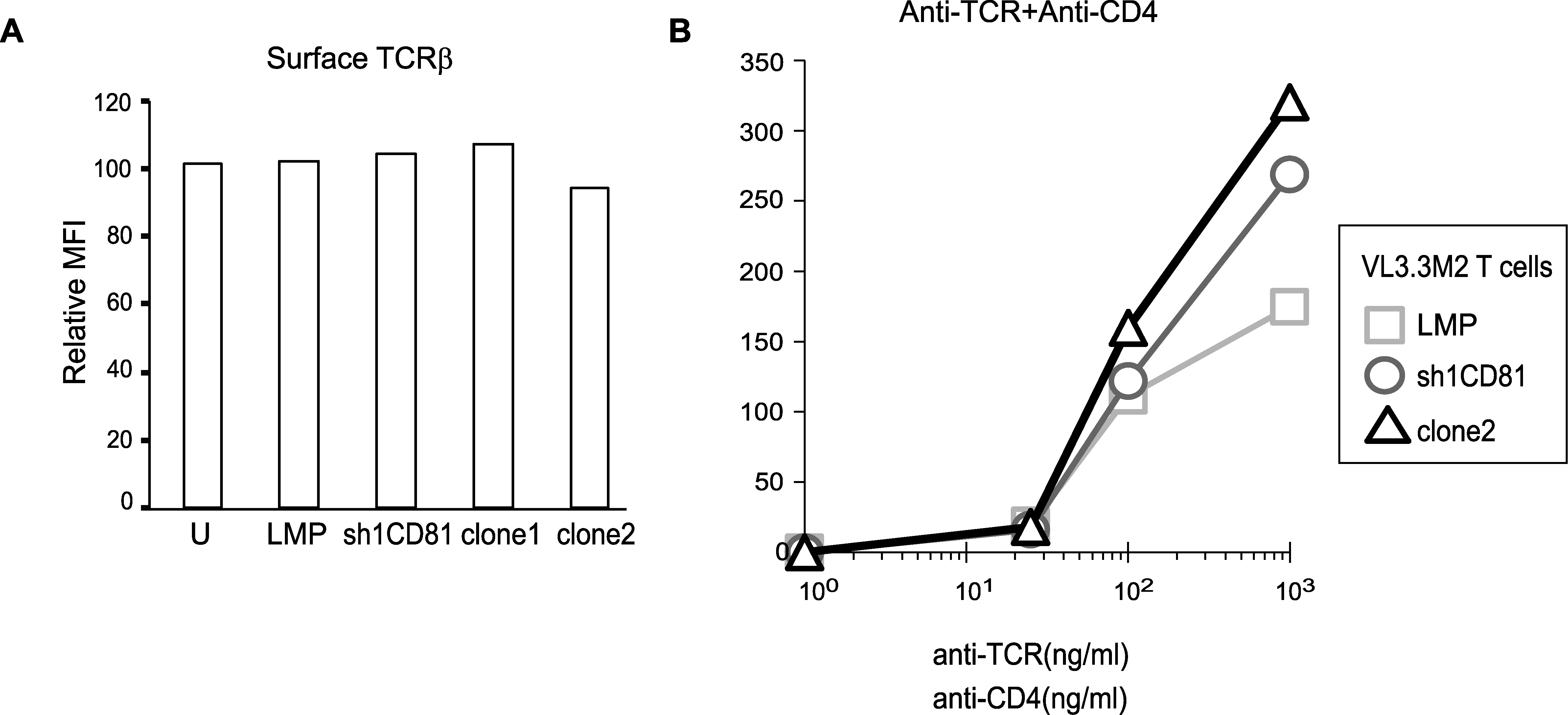

Supplement: Figure S3 — Stable expression of sh1CD81 increases TCR mediated activation without affecting surface TCR expression. (a) CD81 shRNAs does not affect surface TCR expression in stably transfected VL3.3M2 cells. Relative MFI of TCRβ expression on the surface of VL3.3M2 cells that are untransfected (U), or stably transfected with empty pLMP constructs (LMP) or with pLMP-sh1CD81 constructs (sh1CD81) or single cell cloned stable sh1CD81 expressing clones (clone1 and clone2) was determined by flow cytometry and plotted as bar graphs. Surface TCRβ expression of untransfected VL3.3M2 cells was set to 100. (b) Surface CD69 expression activated by anti-TCR+anti-CD4 co-crosslinking is inversely proportional to the level of surface CD81 expression. VL3.3M2 cells were crosslinked with plate bound anti-TCR+anti-CD4 antibodies and MFI of surface CD69 expression on empty LMP transfected (squares), sh1CD81 expressing (circles) and single cell cloned high sh1CD81 expressing clone 2 cells (triangles) were plotted for increasing antibody concentrations. (TIF) [file pone.0050396.s003.tif]

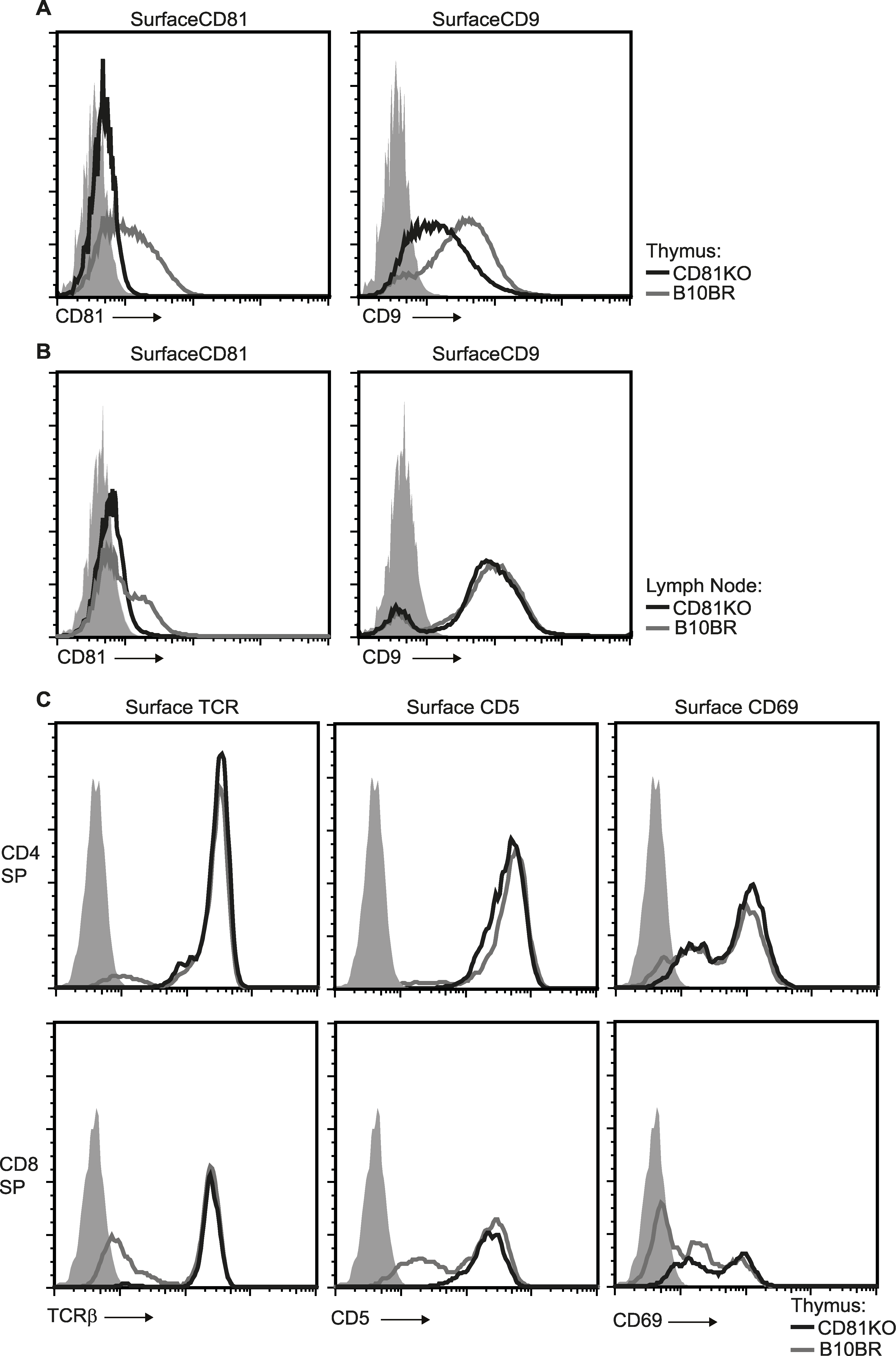

Supplement: Figure S4 — Surface expression of CD81, CD9, TCR, CD5 and CD69 on the surface of CD81−/− and CD9−/− thymocytes and lymph node cells. (a) Surface CD81 and CD9 expression on CD81−/− (black histograms) and CD9−/− (grey histograms) on DP thymocytes shown in the gate defined in Figure 4. Isotype control staining is shown as a shaded histogram. (b) Surface CD81 and CD9 expression on CD81−/− (black histograms) and CD9−/− (grey histograms) on LN cells. (c) Surface TCRβ, CD5 and CD69 expression on CD81−/− (black histograms) and CD9−/− (grey histograms) on CD4 (top row) and CD8 (bottom row) SP thymocytes. (TIF) [file pone.0050396.s004.tif]

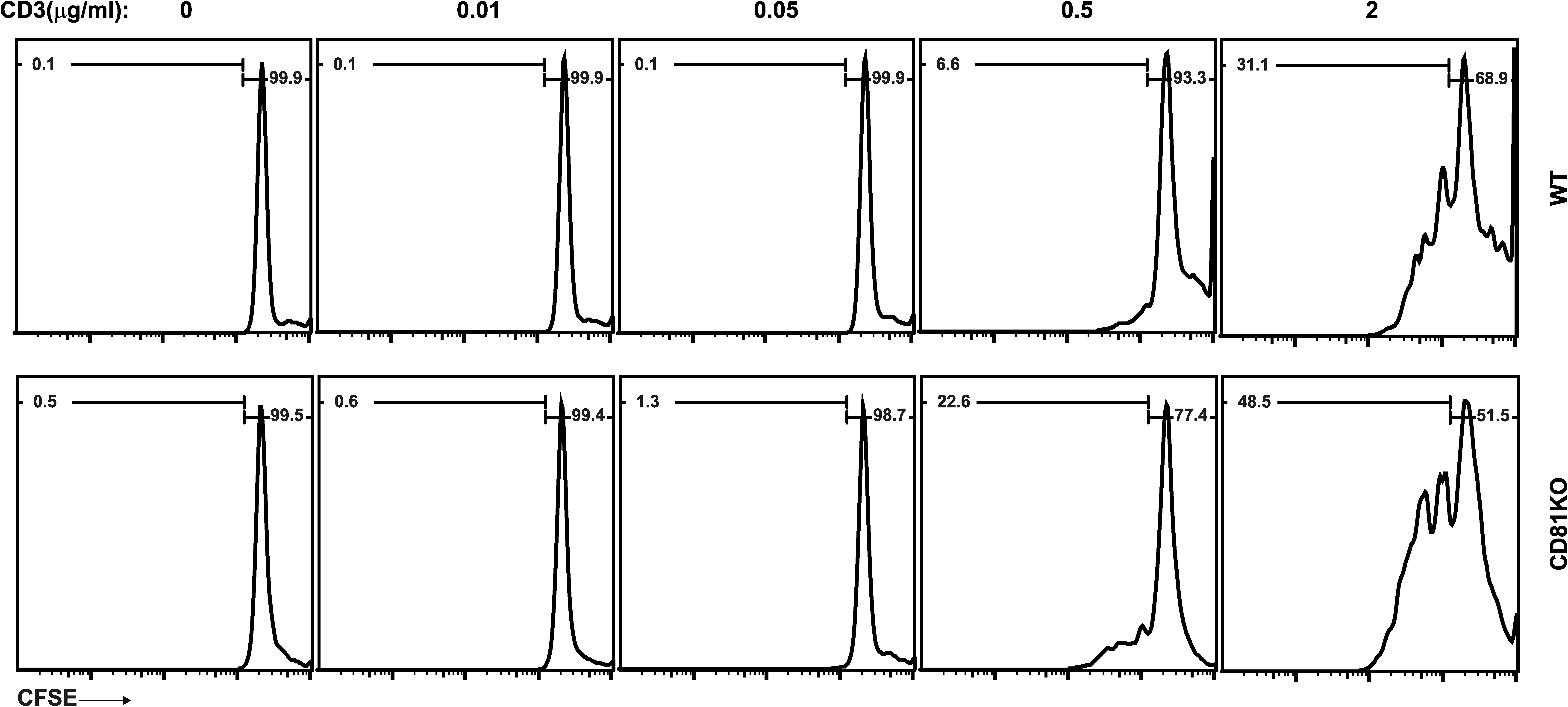

Supplement: Figure S5 — CD81−/− LN CD4+ lymphocytes proliferate faster than WT cells. Frequency of proliferated (>1 cell division) cells after stimulation of CFSE-labeled purified LN CD4+ cells from B6 and CD81−/− mice. Histograms show CFSE expression in stimulated CD4 T cells and numbers in the left gate indicate the frequency of cells with >1 division and the numbers in the right gate indicate un-proliferated cells. (TIF) [file pone.0050396.s005.tif]
